# Abnormal daytime sleepiness in dementia with Lewy bodies compared to Alzheimer’s disease using the Multiple Sleep Latency Test

**DOI:** 10.1186/s13195-014-0076-z

**Published:** 2014-12-10

**Authors:** Tanis J Ferman, Glenn E Smith, Dennis W Dickson, Neill R Graff-Radford, Siong-Chi Lin, Zbigniew Wszolek, Jay A Van Gerpen, Ryan Uitti, David S Knopman, Ronald C Petersen, Joseph E Parisi, Michael H Silber, Bradley F Boeve

**Affiliations:** Department of Psychiatry and Psychology, Mayo Clinic, 4500 San Pablo Road, Jacksonville, FL 32224 USA; Department of Psychiatry and Psychology, Mayo Clinic, 200 First Street SW, Rochester, MN 55905 USA; Department of Pathology, Mayo Clinic, 4500 San Pablo Road, Jacksonville, FL 32224 USA; Department of Neurology, Mayo Clinic, 4500 San Pablo Road, Jacksonville, FL 32224 USA; Center for Sleep Medicine, Mayo Clinic, 4500 San Pablo Road, Jacksonville, FL 32224 USA; Department of Neurology, Mayo Clinic, 200 First Street SW, Rochester, MN 55905 USA; Department of Laboratory Medicine and Pathology, Mayo Clinic, 200 First Street SW, Rochester, MN 55905 USA; Center for Sleep Medicine, Mayo Clinic, 200 First Street SW, Rochester, MN 55905 USA

## Abstract

**Introduction:**

Excessive daytime sleepiness is a commonly reported problem in dementia with Lewy bodies (DLB). We examined the relationship between nighttime sleep continuity and the propensity to fall asleep during the day in clinically probable DLB compared to Alzheimer’s disease (AD) dementia.

**Methods:**

A full-night polysomnography was carried out in 61 participants with DLB and 26 with AD dementia. Among this group, 32 participants with DLB and 18 with AD dementia underwent a daytime Multiple Sleep Latency Test (MSLT). Neuropathologic examinations of 20 participants with DLB were carried out.

**Results:**

Although nighttime sleep efficiency did not differentiate diagnostic groups, the mean MSLT initial sleep latency was significantly shorter in participants with DLB than in those with AD dementia (mean 6.4 ± 5 minutes vs 11 ± 5 minutes, *P* <0.01). In the DLB group, 81% fell asleep within 10 minutes compared to 39% of the AD dementia group (*P* <0.01), and 56% in the DLB group fell asleep within 5 minutes compared to 17% in the AD dementia group (*P* <0.01). Daytime sleepiness in AD dementia was associated with greater dementia severity, but mean MSLT latency in DLB was not related to dementia severity, sleep efficiency the night before, or to visual hallucinations, fluctuations, parkinsonism or rapid eye movement sleep behavior disorder. These data suggest that abnormal daytime sleepiness is a unique feature of DLB that does not depend on nighttime sleep fragmentation or the presence of the four cardinal DLB features. Of the 20 DLB participants who underwent autopsy, those with transitional Lewy body disease (brainstem and limbic) did not differ from those with added cortical pathology (diffuse Lewy body disease) in dementia severity, DLB core features or sleep variables.

**Conclusions:**

Daytime sleepiness is more likely to occur in persons with DLB than in those with AD dementia. Daytime sleepiness in DLB may be attributed to disrupted brainstem and limbic sleep–wake physiology, and further work is needed to better understand the underlying mechanisms.

## Introduction

Daytime somnolence is commonly reported in patients with dementia with Lewy bodies (DLB) [1–3], and it is a major stressor for caregivers [4]. When daytime sleepiness is subjectively and objectively found in AD dementia, it is typically related to greater dementia severity [5,6]. In contrast, DLB daytime sleepiness based on informant report occurs early in the disease [2] and has been documented to occur in the Mild Cognitive Impairment stage of DLB [7]. Using the Multiple Sleep Latency Test (MSLT), we sought to objectively confirm whether patients with DLB have a greater propensity to fall asleep in a permissive setting compared to patients with AD dementia. If daytime sleepiness can be empirically confirmed in early DLB and distinguished from AD dementia, this has implications for the early clinical detection of DLB.

Since nighttime sleep debt is well known to increase the daytime drive to sleep in normal populations [8], we investigated whether sleep fragmentation or poor sleep efficiency the night before was associated with subjective and objective daytime sleepiness. Moreover, sleep fragmentation due to respiratory and movement-related arousals can occur in DLB and Parkinson’s disease [9–12], but it is not known if these nocturnal arousals are sufficient to interfere with daytime alertness.

## Methods

### Patients

Patients were consecutively recruited through the Neurology and Neuropsychology clinics at the Mayo Clinic and enrolled as part of the Mayo Clinic’s Alzheimer’s Disease Research Center (ADRC; Jacksonville, FL, and Rochester, MN, USA). All patients had a reliable informant who provided a clinical history and completed symptom rating scales. The clinical diagnosis was determined by a consensus of neurologists and neuropsychologists. Patients were asked to participate if criteria were met for clinically probable DLB requiring dementia plus at least two of four clinical features (visual hallucinations, fluctuations, parkinsonism and rapid eye movement (REM) sleep behavior disorder (RBD)) [13]. Established diagnostic criteria for clinically probable AD dementia were used [14]. The determination of the presence of dementia was based on formal neurocognitive assessment requiring at least two areas of cognitive impairment and informant report of impaired instrumental activities of daily living that represented a decline from premorbid levels [15]. The terms *dementia with Lewy bodies* and *Alzheimer’s disease dementia* are used to represent clinically probable DLB and AD dementia, respectively.

The study was approved by the Mayo Clinic Institutional Review Board, and informed consent for participation was obtained from every participant and a surrogate.

### Clinical characterization

We administered the Global Deterioration Scale (GLDS) [16] and the Folstein Mini Mental State Examination score [17] to represent general ratings of dementia severity. A history of the presence or absence of RBD was documented with the Mayo Sleep Questionnaire [18] and confirmed via informant interview. Each patient underwent a neurologic examination, which included the Unified Parkinson’s Disease Rating Scale for motor signs [19]. Patients were considered to have parkinsonism if two of the four cardinal features were present (bradykinesia, rigidity, resting tremor and/or postural instability). Fluctuations were deemed present based on a score of 3 or 4 on the Mayo Fluctuations Scale [1]. Informants completed a visual hallucinations questionnaire and were interviewed to obtain information about the presence, type and onset of visual hallucinations. The Epworth Sleepiness Scale (ESS) was administered to the informant, who was asked to rate the patient’s likelihood to fall sleep in eight situations [20]. Measures of depression were obtained from self-report using the Geriatric Depression Scale–Short Form [21] and from informant report using the Neuropsychiatric Inventory Questionnaire–Short Form (NPI-Q) [22].

### Procedures

A full-night polysomnography was carried out with 61 patients with DLB and 26 patients with AD dementia. The MSLT was undertaken with 32 DLB and 18 AD dementia, and there was no difference in demographics, dementia severity or core features from those who chose not to carry out the MSLT.

The MSLT was comprised of four daytime nap times with 2 hours of wakefulness between each nap opportunity. Participants were asked to lie comfortably with lights out and were advised to try to fall asleep. Sleep onset was noted to occur when there were either three complete epochs of stage 1 sleep or any one epoch of unequivocal sleep. Once either sleep criterion was observed, the initial sleep latency was recorded, the subject was awakened and that nap session was terminated. If there was no sleep onset within a 20-minute period, then the nap session was terminated.

Scoring of sleep stages was carried out according to standard guidelines [23,24], and each polysomnogram was reviewed by a sleep medicine clinician certified by the American Board of Sleep Medicine. All polysomnography studies involved continuous videotaping synchronized to standard monitoring using the following montage: two electro-oculogram derivations, three electroencephalography derivations f(Fz-Cz, Cz-Oz, C4-A1), an electrocardiogram, chin and at least two limb surface electromyographic electrodes, oronasal airflow, sonogram, oxyhemoglobin saturation, and chest and abdomen inductance plethysmography.

*Sleep efficiency* was defined as the total sleep time divided by the total time in bed multiplied by 100%. An *arousal* was defined as an abrupt shift of electroencephalography frequency including alpha, theta or frequency higher than 16 Hz, but not sleep spindles, after at least 10 seconds of stable sleep that lasted at least 3 seconds during any sleep stage, but not long enough to be classified as awake. An arousal during REM sleep was scored only if it was accompanied by increased amplitude of submental electromyography. For an obstructive event, there was a 10-second or longer period with a clear amplitude decrease in breathing from baseline associated with over 3% oxygen desaturation or an arousal from the obstructive events. In a central event, there was a reduction or absence of breathing and respiratory effort that lasted 10 seconds or longer with associated reduced airflow. For a hypopnea, there was a reduction of airflow and a reduction of thoracic and/or abnormal movement that led to an arousal. The respiratory disturbance index represents the sum of disordered breathing events related to obstructive apneas, central apneas, mixed apneas, hypopneas and respiratory effort–related changes averaged over the total sleep time, which represents a value of the sum per hour. A *periodic limb movement* was defined as periodic contraction of the lower legs, either unilateral or bilateral, with a series of four consecutive movements separated by 4 to 90 seconds, with each movement lasting between 0.5 and 5 seconds and not associated with respiratory events. The mean number of periodic limb movements per hour associated with arousals was considered the movement-related arousals per hour. A *spontaneous arousal* was defined as an arousal not related to disordered breathing or movements, and the spontaneous arousal per hour reflects the number of spontaneous arousals per hour averaged over total sleep time. The finding of REM sleep without atonia was considered present if muscle tone during REM sleep was unequivocally abnormally increased and if no epileptiform discharges were noted on the record.

### Neuropathological examination

Autopsy specimens were obtained for 20 patients with DLB and none of the patients with AD. Standardized neuropathologic assessments, including macroscopic and microscopic evaluations, were carried out with assignment of a pathologic diagnosis using established DLB criteria [13,25]. Lewy body distribution was determined on the basis of Lewy body counts using a polyclonal antibody to α-synuclein, with diffuse Lewy body disease (DLBD), including those with Lewy-related pathology in the neocortex and limbic and brainstem regions, and transitional Lewy body disease (TLBD), including those with Lewy-related pathology in the limbic and brainstem regions. Braak neurofibrillary tangle (NFT) stage was identified using thioflavin-S microscopy or the Bielschowsky silver stain technique [26].

### Statistical analysis

For each patient group, sleep efficiency and mean MSLT initial sleep latency showed normal distributions using the Kolmogorov-Smirnov test. Equality of variance was confirmed using the Levene test of homogeneity of variance and Mauchly’s test of sphericity. Comparisons of continuous variables used the one-way analysis of variance, and comparisons of categorical variables used the Chi-square test. A repeated measures analysis of covariance was used to compare the four MSLT nap latencies between DLB and AD dementia with the GLDS (a measure of dementia severity) as the covariate. Two-tailed Pearson correlational analyses were carried out to examine the associations between continuous variables. In an effort to reduce type 1 error from multiple comparisons, the *P*-value for significance was set at ≤0.01. To determine how nighttime sleep efficiency compared to community-dwelling older adults without an established dementia, individual *z*-scores were calculated based on data stratified by age and sex in a large community sample [27].

## Results

### Clinical characterization

In the DLB group, 23% had two core DLB features, 41% had three core DLB features and 36% had four core DLB features. In the AD dementia group, eight participants had one of the core DLB features. Demographic and clinical variables were compared between groups (see Table 1). The patient groups did not differ in age, education, dementia severity or duration of cognitive impairment. Parkinsonism severity, based on UPDRS scores, was greater in DLB compared to AD dementia. Informants provided higher ratings on the Epworth Sleepiness Scale for DLB compared to AD dementia. The DLB group had higher self-reported depression scores compared to the AD dementia group, though there was no difference in informant report of depression between groups using the NPI-Q. There was no difference between DLB and AD dementia in the frequency of cholinesterase inhibitor use (DLB 73% vs AD dementia 62%, *X*^2^ = 1.2, p =0.27). Of the DLB group, 36% were taking carbidopa-levodopa. Of the four DLB patients prescribed pramipexole or ropinirole, two were also taking carbidopa-levodopa. In the AD dementia group, 7% were taking carbidopa-levodopa. None of the patients were taking amantadine, anticholinergic agents, or benzodiazepines at the time of the sleep study. There was no difference in demographics, dementia severity, number or duration of core DLB features between patients who participated in the MSLT compared to those who opted out.

**Table 1:** **Demographic and clinical variables** ^**a**^

**Variables**	**DLB**	**AD dementia**	***F*** **/** ***χ*** ^**2**^	***P*** **-value**
No. of participants	61	26		
Males, %	84%	62%	5.0	0.03
Age, yr	70.5 ± 7	70.8 ± 8	0.04	0.84
Education, yr	14.6 ± 3	14.8 ± 3	0.12	0.73
Estimated duration of cognitive impairment, yr	3.6 ± 2	4.6 ± 4	2.4	0.12
Mini Mental State Examination score	23.4 ± 5	23.4 ± 4	0.004	0.95
Global Deterioration Scale score	3.9 ± 1	3.7 ± 1	0.44	0.51
Visual hallucinations, %	59%	8%	19.5	<0.01
Estimated duration of visual hallucinations, yr	2.1 ± 2	2.0 ± 1	0.008	0.93
Parkinsonism, %	84%	0%	–	–
Estimated duration of parkinsonism, yr	2.7 ± 3	–	–	–
Unified Parkinson’s Disease Rating Scale score	10.4 + 8	0.35 + 1	29.0	<0.01
Probable RBD, %	90%	3.8%	–	–
Estimated duration of RBD, yr	10.2 ± 11	2	–	–
Mayo Fluctuations Scale score >2, %	81%	21%	26.7	<0.01
Epworth Sleepiness Scale, informant	13.0 ± 6	8.3 ± 5	11.4	<0.01
Geriatric Depression Scale Score	4.6 ± 3.8	2.0 ± 1.5	9.3	<0.01
NPI-Q depression, informant report, %	36%	30%	0.19	0.66

^a^Values represent mean ± standard deviation, except where indicated otherwise. AD dementia, Alzheimer’s disease dementia; DLB, Dementia with Lewy bodies; NPI-Q, Neuropsychiatric Inventory Questionnaire–Short Form; RBD, Rapid eye movement sleep behavior disorder. We used Fisher’s exact test in χ^2^ comparisons with less than five per cell, and no statistical comparisons were made with one or less per cell.

### Full-night polysomnography

Overnight polysomnography data are presented in Table 2. There was no difference in total sleep time, sleep efficiency or arousals during sleep between the DLB and AD dementia groups. There was also no difference in the number or types of arousals per hour or in the percentage of sleep time affected by arousals between groups. Arousals from periodic limb movements did not distinguish groups and were relatively infrequent, with a movement-related arousal index ≥15 in 8% of the DLB group and in 15% of the AD group (*χ*^2^ = 0.95, *P* =0.44). Breathing-related arousals with a respiratory disturbance index ≥15 were found 18% of the DLB group and 15% of the AD dementia group and did not differentiate groups (*χ*^2^ = 0.11, *P* =0.74).

**Table 2:** **Overnight polysomnography in the dementia with Lewy bodies and Alzheimer’s disease dementia groups** ^**a**^

	**DLB**	**AD dementia**	***F*** **/** ***χ*** ^**2**^	***P*** **-value**
No. of participants	61	26		
Time in bed, min	488 ± 78	560 ± 65	17	<0.01
Total sleep time, min	348 ± 99	382 ± 77	2.43	0.12
Sleep efficiency, %	72 ± 18	69 ± 17	0.24	0.63
Initial sleep latency, min	27 ± 38	23 ± 20	0.26	0.61
Initial REM sleep latency, min	144 ± 101	140 ± 105	0.03	0.87
Stage N1, % total sleep time	12 ± 11	16 ± 12	1.9	0.17
Stage N2, % total sleep time	53 ± 16	53 ± 13	0.005	0.99
Stage N3, % total sleep time	22 ± 16	14 ± 9	5.6	0.02
Stage REM, % total sleep time	14 ± 12	17 ± 8	1.7	0.19
Arousal index/hr	25 ± 15	29 ± 17	0.88	0.35
Respiratory disturbance index/hr	10 ± 11	8 ± 12	0.55	0.46
Respiratory disturbance index ≥15, %	18%	15%	0.11	0.74
Movement-related arousals/hr	4.3 ± 7	5.8 ± 6	1.28	0.28
Spontaneous arousals/hr	8.7 ± 8	9.8 ± 8	0.32	0.57
Oxygen saturation, %	94 ± 2	95 ± 2	0.52	0.47
REM sleep without atonia, %	71%	8%	43.4	<0.01
Absence of REM sleep, %	19%	0%	–	–

^a^Values represent mean ± standard deviation or percentage per group. Index values/hour reflect the number of arousals per total hours of sleep time. AD dementia, Alzheimer’s disease dementia; DLB, Dementia with Lewy bodies; REM, Rapid eye movement. We used Fisher’s exact test *for χ* ^2^ comparisons with less than five per cell, and no statistical comparisons were made with one or less per cell.

In the DLB group, there was no relationship between nighttime sleep efficiency and age, dementia severity, sex, fluctuations, parkinsonism severity, depression indices or the presence or duration of visual hallucinations, RBD or parkinsonism. When compared to age- and sex-stratified normative data for older adults [27], mean nighttime sleep efficiency was average for the DLB group (mean *z*-score = −0.6 ± 1.7) and low average for the AD dementia group (mean *z*-score = −1.1 ± 1.6). In DLB, poor sleep efficiency was associated with longer nighttime initial sleep latency (*r* = −0.41, *P* <0.01), more time in N1 (r = −0.52, *P* <0.01), a greater arousal index (*R* = −0.33, *P* =0.01) and more spontaneous arousals (*r* = −0.37, *P* <0.01). Poor sleep efficiency in DLB was not associated with informant report of daytime sleepiness based on the Epworth Sleepiness Scale.

In the AD dementia group, there was no relationship between nighttime sleep efficiency and age, sex, dementia severity, fluctuations, parkinsonism severity or depression indices. Poor sleep efficiency was associated with more time in N1 (*r* = −0.65, *P* <0.01) and a greater arousal index (*r* = −0.60, *P* <0.01). Poor sleep efficiency in AD dementia showed a nonsignificant trend with informant report of daytime sleepiness based on the Epworth Sleepiness Scale (*r* = −0.43, *P* <0.04).

Use of a cholinesterase inhibitor was not associated with differences in clinical, demographic or sleep variables between the diagnostic groups. Patients with DLB who were taking carbidopa-levodopa had greater parkinsonism severity compared to those not taking this agent (mean UPDRS =15 ± 7 vs 8 ± 7, *F* =13, *P* <0.01), but they also had a lower arousal index (mean arousal index =19 ± 11 vs 29 ± 17, *F* =6.2, *P* =0.01) and fewer spontaneous arousals (mean spontaneous arousal/hour =5 ± 5 vs 11 ± 9, *F* =8.3, *P* <0.01), suggesting a sleep benefit associated with such treatment. This effect did not change when the few patients taking dopamine agonists were included in the comparison.

A clinical history of RBD was present in 90% of the DLB group. REM sleep without atonia was confirmed in 71%, but REM sleep was not achieved in 19%, rendering it impossible to formally confirm RBD for them. In this group with a clinical history of RBD, the patients who did not achieve REM sleep had significantly less total sleep time (no REM sleep =265 ± 99 minutes vs REM sleep without atonia =370 ± 91 minutes, *F* =11.2, *P* <0.01) and lower sleep efficiency (no REM sleep =58% ±19 vs REM sleep without atonia =75% ± 16, *F* =12.3, *P* <0.01) than their counterparts who achieved REM sleep. REM sleep without atonia was found in two patients with AD dementia, despite the absence of a clinical history of dream enactment behavior during sleep.

### Multiple Sleep Latency Test

**Figure 1:**
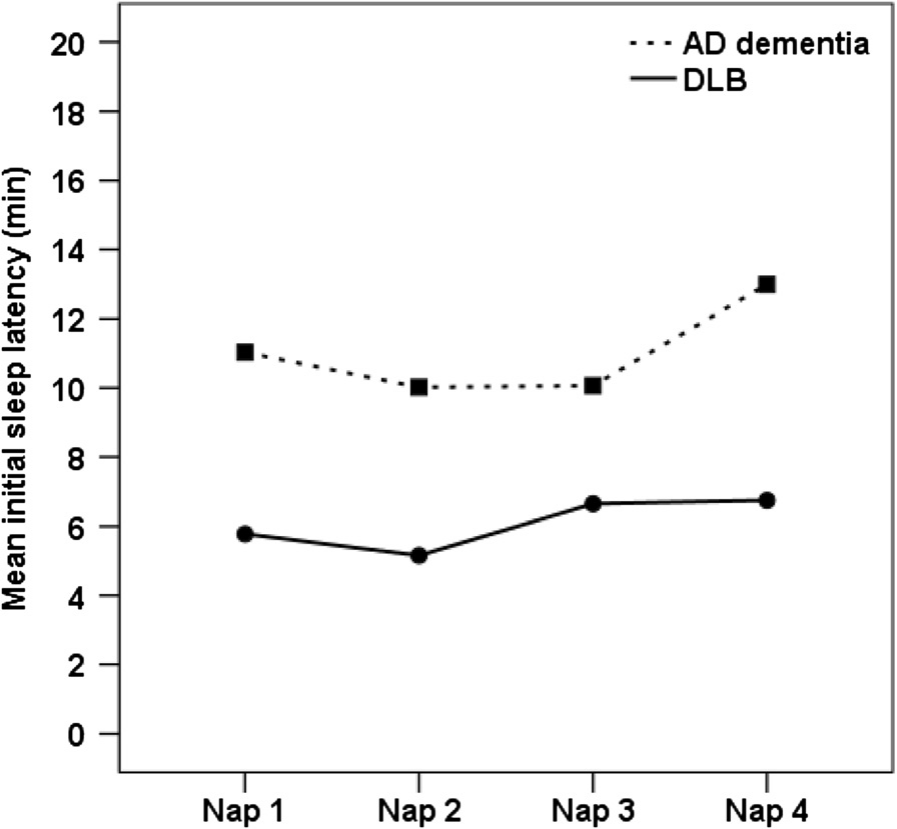
**Multiple Sleep Latency Test mean initial sleep latency in dementia with Lewy bodies and Alzheimer’s disease dementia groups.** AD, Alzheimer’s disease; DLB, Dementia with Lewy bodies.

Patients with DLB were more likely than those with AD dementia to have an abnormal mean MSLT initial sleep latency <10 minutes (DLB =81% vs AD dementia =39%, *χ*^2^ = 9.2, *P* <0.01) and <5 minutes (DLB =56% vs AD dementia =17%, *χ*^2^ = 7.4, *P* =0.01). The mean MSLT initial sleep latency was shorter for DLB than AD dementia (DLB =6.4 ± 5 minutes vs AD dementia =11.3 ± 5 minutes, *F* =12.6, *P* <0.01). Since dementia severity was associated with shorter mean MSLT initial sleep latencies in the AD dementia group (*r* = −0.59, *P* <0.01), we also carried out a repeated measures analysis of covariance with GLDS as the covariate. The results showed a significant between-subjects effect, confirming shorter MSLT mean initial sleep latencies across the four naps for the DLB group compared to the AD dementia group (*F* =14.5, *P* <0.001). There were no within-subjects effects, indicating no differences in mean initial sleep latencies between each of the four nap opportunities for either dementia group (see Figure 1).

In DLB, mean MSLT initial sleep latency was not associated with age, dementia severity, sex or use of antiparkinsonian agents or cholinesterase inhibitors. There was also no relationship with clinical variables, such as the number of core DLB features, parkinsonism severity or duration of visual hallucinations, parkinsonism or RBD. Informant ratings of fluctuations and Epworth Sleepiness Scale items were correlated in the DLB group (*r* =0.45, *P* <0.01) and in the AD dementia group (*r* =0.62, *P* <0.01), but these data did not reach statistical significance when compared with mean MSLT initial sleep latency. In DLB, nighttime sleep efficiency and arousal index were unrelated to mean MSLT initial sleep latency (*r* = −0.05, *P* =0.78), indicating that daytime sleepiness occurred regardless of the degree of nighttime sleep fragmentation. When the sample was limited to include only the very mild and mild stage of dementia, the differences between the DLB and AD dementia groups in informant Epworth Sleepiness Scale scores and mean MSLT initial sleep latencies were upheld. In the AD dementia group, there was a trend in the relationship between nighttime sleep efficiency and mean MSLT initial sleep latency (*r* = −0.49, *P* <0.04) with a subset who had trouble sleeping at night who were also less likely to sleep during the day. This type of low sleepability and hyperarousability is well documented in primary insomnia [28,29], but our study did not have sufficient statistical power for us to examine this relationship further in our AD dementia cohort.

### Neuropathologic characterization

In the DLB group, 20 patients came to autopsy an average of 4.1 ± 2 years following the formal sleep study. All had neuropathologic confirmation of intermediate or high likelihood DLB. Among these patients, eight had TLBD with predominantly brainstem/subcortical Lewy-related pathology and twelve had DLBD with additional cortical Lewy-related pathology. In the TLBD group, five had a Braak NFT stage less than IV and three had a Braak NFT stage of IV. In the DLBD group, two had a Braak NFT stage less than IV, seven had a Braak NFT stage of IV and three had a Braak NFT stage greater than IV.

Braak NFT stage, Lewy distribution (TLBD vs DLBD) and intermediate vs high likelihood DLB were not associated with demographic, clinical, sleep or dementia severity indices. As such, widespread cortical pathology does not appear to be a requirement for the dementia or other clinical or sleep features of DLB, including subjective or objective daytime sleepiness.

## Discussion

Patients with DLB exhibited greater daytime somnolence than those with AD dementia of similar age, sex and dementia severity. On the MSLT, 81% of the DLB group fell asleep within a mean of 10 minutes in four daytime nap opportunities, compared to 39% of the AD group. Pathologic sleepiness, based on a mean MSLT initial sleep latency of less than 5 minutes, was evident in 56% of the DLB group compared to 17% of the AD dementia group. This was consistent with subjective informant ratings of a higher Epworth Sleepiness Scale score in DLB compared to AD dementia from the larger sample. Although daytime somnolence was associated with dementia severity in AD dementia, this was not the case in DLB. These data empirically confirm that daytime sleepiness is more likely to occur in patients with DLB than in those with AD dementia.

In DLB, the increased propensity to fall asleep during the day was not related to poor sleep quality the night before. Mean nighttime sleep efficiency was average when individual scores were compared to published age- and sex-stratified norms [27]. Moreover, nighttime sleep efficiency in the DLB group was not associated with daytime sleepiness based on either objective measurement or informant ratings. Nonetheless, since poor sleep efficiency in the DLB group was associated with a higher number of spontaneous arousals, we examined whether extrapyramidal features, such as motor stiffness, which can disrupt sleep by restricting one’s ability to turn over [30], might have contributed to the daytime somnolence in our DLB group. This has particular relevance, because daytime sleepiness is often seen in persons with Parkinson’s disease [31–36]. In our sample, the patients with DLB who had more severe motor problems actually had fewer spontaneous arousals, a sleep benefit related to their use of carbidopa-levodopa. In addition, parkinsonism severity or use of carbidopa-levodopa was not associated with objective and subjective measures of daytime sleepiness. Therefore, neither parkinsonism severity nor carbidopa-levodopa appears to be primarily responsible for the daytime somnolence in our DLB sample.

Sleep disorders, such as moderate to severe sleep apnea and periodic limb movement-related arousals, occurred in less than 20% of the entire sample, which is consistent with rates expected in normal community-dwelling older adults [37–39]. Although 81% of the DLB group had a mean MSLT initial sleep latency shorter than 10 minutes, nighttime arousals related to respiration or movement were not associated with mean MSLT initial sleep latency or with informant ratings of the Epworth Sleepiness Scale of the larger sample. Thus, the presence of these sleep disorders did not account for disturbed arousal in our DLB group.

In our DLB cohort, objective and subjective assessment of an individual’s susceptibility to fall asleep during the day did not depend on the presence or duration of visual hallucinations, parkinsonism or RBD. Although the informant ratings of DLB fluctuations and sleepiness were interrelated, they were not perfectly correlated, and the correlation between DLB fluctuations and mean MSLT initial sleep latency did not reach significance. This suggests that despite some overlap between DLB fluctuations and sleepiness, they are distinct entities. A similar relationship has been observed in delirium, where altered consciousness is an important contributor but other signs must be present for a diagnosis of delirium to be made [40,41]. Likewise, although disturbed arousal is a consistent element of DLB fluctuations [1,42], the added presence of other components, such as inconsistent abilities, episodes of incoherent speech or variable attention, are needed to constitute the waxing and waning state that characterize DLB fluctuations [1,42–45]. Taken together, our data provide evidence that daytime sleepiness is a distinct feature of DLB that is not contingent on disease stage or on any one of the four core features of DLB. Further work is needed to determine if its presence helps improve diagnostic validity and reliable early detection of DLB.

Of the 20 patients with DLB who underwent pathologic examination, all had confirmation of Lewy body disease. There was no difference in demographics, clinical or sleep variables between the eight patients with TLBD (which includes brainstem and limbic Lewy pathology) and the twelve with DLBD (includes added cortical pathology). Similarly, there was no difference between those with intermediate likelihood DLB and high likelihood DLB, which also takes into account concomitant neurofibrillary tangle pathology. As such, neuronal loss and Lewy pathology in brainstem and limbic regions, without widespread cortical involvement, are sufficient to produce daytime sleepiness, dementia and the other core DLB features. This is consistent with the Braak staging model of Lewy body disease, which suggests earlier involvement of brainstem and limbic regions relative to cortical regions [46].

We postulate that the mechanism underlying daytime sleepiness in DLB may be related to neuronal loss from the disease itself and triggered by the disruption of the brain regions responsible for sleep–wake physiology. In Lewy body disease, cell clusters that are particularly vulnerable include the locus coeruleus, the raphe nucleus, the tuberomammillary nucleus of the hypothalamus, the periaqueductal gray and the basal forebrain [25,47]. These nuclei constitute a neuronal network composed of multiple neurotransmitters known to regulate wakefulness that are referred to collectively as the ascending reticular activating system (ARAS) [48–52]. Saper and colleagues [53] have proposed a sleep–wake switch model based on the reciprocal relationship between the wakefulness neurons of the ARAS and the sleep neurons of the ventrolateral preoptic hypothalamus (VLPO) [53], with the hypocretin cells of the lateral hypothalamus serving as a modulator of sleep–wake transitions [54,55]. Further work is needed to determine whether our finding of essentially normal nighttime sleep efficiency in DLB, but greater daytime sleepiness, may reflect a bias or imbalance between the VLPO and ARAS, and whether there is an inequity in their modulation by the lateral hypothalamic hypocretin neurons. Although cerebrospinal fluid levels of hypocretin in DLB and Parkinson’s disease dementia show wide variability, ranging from very low to normal levels [56–58], there is pathologic evidence of greater hypocretin immunoreactive cell loss in DLB compared to AD [59,60], with one DLB study showing hypocretin cell loss correlating with hypersomnolence and α-synuclein [60]. Whether hypocretin cell loss is directly related to Lewy-related pathology or is a consequence of lost input due to damage to the ARAS neuronal network in Lewy body disease is not yet known. More detailed investigation of how the particular pathways known to be involved in sleep and wakefulness are affected in DLB is clearly needed.

A clinical history of RBD was present in 90% of our DLB sample, but it could be confirmed in only the 71% who actually achieved REM sleep during polysomnography. Of the 19% with DLB and a history of RBD who did not achieve REM sleep, these patients had less total sleep time and lower nighttime sleep efficiency than their counterparts who did achieve REM sleep. Those with a longer documented clinical duration of RBD also spent less time in REM sleep, which may explain why RBD eventually becomes quiescent in patients with very long histories of RBD [61].

Some limitations to the study deserve mention. Replication with a larger sample size and with complete congruence between those who have undergone overnight polysomnography and daytime MSLT is needed. In addition, interpretation of the neuropathologic analysis is limited by the absence of AD autopsies and by the small number of DLB cases with sleep evaluations who have come to autopsy to date. In our effort to determine whether nighttime sleep efficiency was normal for age and sex, we calculated individual *z*-scores using a large normative data set that incorporated in-home overnight polysomnography. Although this setting may not be exactly comparable to the sleep laboratory, studies indicate good validity with in-home polysomnography, and discrepancies tend to be in the direction of better sleep efficiency at home relative to the sleep laboratory [62,63]. Under these conditions, we deemed it reasonable to provide this comparison, recognizing that our laboratory findings of average sleep efficiency in the DLB group and low average sleep efficiency in the AD group may reflect an underestimate of true sleep efficiency for these groups.

In this study, we incorporated the MSLT, which is considered the gold standard for the objective measurement of sleepiness and relies on the assessment of how quickly one falls asleep when asked to do so. Further study is needed to investigate whether patients with DLB also have trouble maintaining wakefulness when asked to do so.

## Conclusions

This study provides objective confirmation, based on polysomnography data, that excessive daytime sleepiness is more likely to occur in patients with DLB than in those with AD dementia and that it is not attributable to poor sleep the night before. Moreover, daytime sleepiness in patients with DLB occurs in the early stages of the disease, whereas it tends to be associated with greater dementia severity in patients with AD dementia. These data provide evidence that daytime sleepiness can be distinguished from the other core DLB features, including fluctuations. If daytime sleepiness is a unique clinical feature of DLB, this has implications for improved early detection and differential diagnosis of DLB, for consideration of alternate treatment interventions, and for promoting our understanding of the pathologic and neuroanatomic involvement in DLB.

## Note

This article is part of a series on Lewy Body Dementia, edited by Ian McKeith and James Galvin. Other articles in this series can be found at http://alzres.com/series/LewyBodyDementia

## Abbreviations

AD: Alzheimer’s disease
ARAS: Ascending reticular activating system
DLBs: Dementia with Lewy bodies
DLBD: Diffuse Lewy body disease
ESS: Epworth Sleepiness Scale
GLDS: Global Deterioration Scale
MMSE: Mini Mental State Examination
MSLT: Multiple Sleep Latency Test
NFT: Neurofibrillary tangle
NPI-Q: Neuropsychiatric Inventory Questionnaire–Short Form
RBD: Rapid eye movement sleep behavior disorder
RDI: Respiratory disturbance index
REM: Rapid eye movement
TLBD: Transitional Lewy body disease
UPDRS: Unified Parkinson’s Disease Rating Scale
VLPO: Ventrolateral preoptic hypothalamus
